# Potential New Inflammatory Markers in Bronchiectasis: A Literature Review

**DOI:** 10.3390/cimb46070398

**Published:** 2024-06-29

**Authors:** Francesco Rocco Bertuccio, Nicola Baio, Simone Montini, Valentina Ferroni, Vittorio Chino, Lucrezia Pisanu, Marianna Russo, Ilaria Giana, Alessandro Cascina, Valentina Conio, Amelia Grosso, Erica Gini, Federica Albicini, Angelo Guido Corsico, Giulia Maria Stella

**Affiliations:** 1 Unit of Respiratory Disease, Cardiothoracic and Vascular Department, IRCCS Policlinico San Matteo, Viale Golgi 19, 27100 Pavia, Italy; francesco.bertuccio01@gmail.com (F.R.B.); nicola.baio01@universitadipavia.it (N.B.); simone.montini01@universitadipavia.it (S.M.); valentina.ferroni01@universitadipavia.it (V.F.); lucrezia.pisanu01@universitadipavia.it (L.P.); marianna.russo01@universitadipavia.it (M.R.); ilaria.giana01@universitadipavia.it (I.G.); a.cascina@smatteo.pv.it (A.C.); v.conio@smatteo.pv.it (V.C.); a.corsico@smatteo.pv.it (A.G.C.); 2 Department of Internal Medicine and Pharmacology, University of Pavia, 27100 Pavia, Italy; a.grosso@smatteo.pv.it (A.G.); erica.gini@gmail.com (E.G.); federica.albicini@gmail.com (F.A.); 3 Ospedale Pederzoli, Peschiera del Garda, 37121 Verona, Italy; vittorio.chino01@universitadipavia.it

**Keywords:** bronchiectasis, inflammatory molecular drivers, TSLP, mucins, endotype, personalised medicine

## Abstract

Specific molecular and inflammatory endotypes have been identified for chronic respiratory disorders, including asthma and COPD (chronic obstructive pulmonary disease). These endotypes correspond with clinical aspects of disease, enabling targeted medicines to address certain pathophysiologic pathways, often referred to as “precision medicine”. With respect to bronchiectasis, many comorbidities and underlying causes have been identified. Inflammatory endotypes have also been widely studied and reported. Additionally, several genes have been shown to affect disease progression. However, the lack of a clear classification has also hampered our understanding of the disease’s natural course. The aim of this review is, thus, to summarize the current knowledge on biomarkers and actionable targets of this complex pathologic condition and to point out unmet needs, which are required in the design of effective diagnostic and therapeutic trials.

## 1. Introduction

Bronchiectasis is a heterogenous chronic respiratory disease characterised by irreversible dilatation of the bronchial tree, excessive mucus production, chronic inflammation, and airway thickening, all of which make it difficult to eliminate sputum; clinically, its primary features are cough, excessive sputum production, and frequent respiratory infections [1]. The airways of patients affected by bronchiectasis present, in the most severe forms, a chronic inflammatory state. Numerous hereditary disorders (Table 1) and a wide range of acquired conditions, such as post-infective conditions, can be the cause of bronchiectasis. However, in 40% of cases, the cause of the illness remains unknown despite thorough diagnostic investigation, identifying bronchiectasis as “idiopathic” [2,3].

Worldwide variations exist in bronchiectasis’ prevalence, underlying aetiology, and presentation, among other factors. The prevalence is rising globally, in Europe, and in the United Kingdom. There has historically been latency between the onset of symptoms and official diagnosis, despite this topic being highlighted frequently. The pathophysiology of bronchiectasis is based on a vicious cycle that includes inflammation, airway infection, decreased mucociliary clearance, and structural damage that accelerates the disease’s progression. Heterogeneity is a well-known characteristic of bronchiectasis [5]. Certain conditions, such as higher than three exacerbations per year, the presence of a chronic *Pseudomonas aeruginosa* infection, and the presence of multiple comorbidities, are linked to greater and faster degradation of clinical status and a significant reduction in the quality of life of patients affected by bronchiectasis [6,7].

Epidemiologically, a recent Italian study has shown a notable rise in the prevalence of bronchiectasis disease from 62 to 163 cases per 100,000 inhabitants between 2005 and 2015 [8]. In addition, a higher prevalence of the disease has been observed in individuals of the female sex (178 cases per 100,000) or older adults (up to 75 years old, or 497 cases per 100,000 residents). The rise in the incidence and prevalence of bronchiectasis should be considered as a warning sign due to the association between the illness and higher mortality, higher hospitalisations, and lower quality of life for bronchiectasis patients relative to the general population [9]. In recent years, research and analysis on this pathology have focused more on the underlying molecular and physio pathological mechanisms, collectively called “endotyping”. An endotype is a subtype of a pathological condition that is identified by a specific physio pathologic mechanism. Novel biological investigations to find disease biomarkers and their correlations have been made possible by new biological technologies like transcriptomics, proteomics, and genomics as well as more advanced bioinformatics and biostatistics applications. This type of analysis could identify tailored therapies that are specific to the patient’s type and individualize for the underlying physio pathological mechanism. It follows that it is essential to identify potential inflammatory drivers [10].

One example of this approach is the use of biological drugs for asthma, which has revolutionized its treatment, especially in severe forms. Among the various agents used in the treatment of severe asthma, in 2021, the use of Tezepelumab (an anti-TSLP antibody) was approved and has shown promising results in treating severe asthma that is both TH-2 and non-TH-2 [11]. Furthermore, a study that examine potential relationships between a patient affected by COPD and the levels of serum TSLP was conducted in 2022. The analysis suggests that TSLP could be useful as a prognostic biomarker and another potential therapeutic strategy in COPD patients [12].

Because bronchiectasis is a heterogenous condition, treatment is individualized to support a pathophysiological, symptom–burden approach instead of a pan-syndrome approach. Treatment for bronchiectasis must consider the traits that many patients share with other respiratory conditions. Reviewing various endotypes, phenotypes, microbiology, and overlapping disorders is crucial since it enables more individualized treatment. It is critical to identify the inflammatory triggers and investigate the ciliary phenotypes that are related with them.

Fortunately, bronchiectasis is gaining attention and research activity; moreover, considering that cytokine-promoted inflammation plays a crucial role in this chronic disease, it may also become one of the hallmarks of the era of immune-modulatory treatments.

In this study, we focus on the potential role of TSLP and mucins as potential new markers in bronchiectasis.

## 2. Thymic Stromal Lymphopoietin (TSLP)

Bronchial epithelial cells release alarmins, a subgroup of cytokines, in reaction to endogenous or exogenous danger signals; alarmins engage in the Th2 inflammatory cascade—among them, thymic stromal lymphopoietin (TSLP) has gained attention in recent years. A less extensive investigation was conducted on the clinical consequences related to the levels of alarmins.

**Figure 1:**
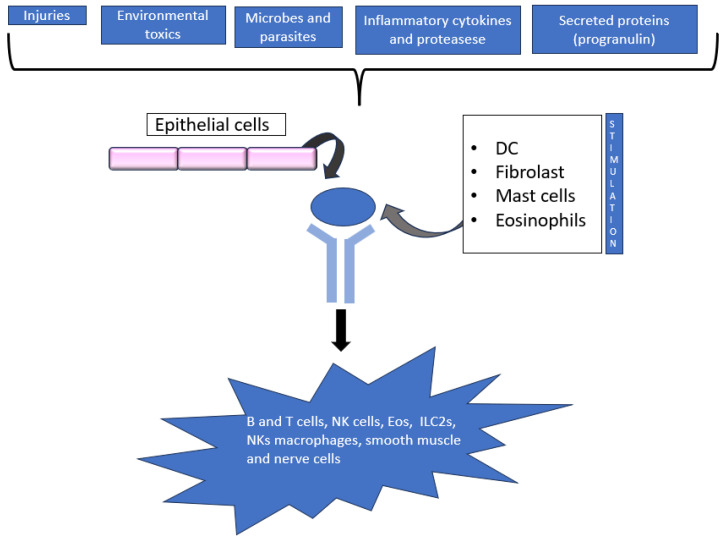
TSLP signalling.

Biologics targeting the alarmins have gained increased attention, especially after the anti-TSLP antibody Tezepelumab demonstrated potential in patients with severe, uncontrolled asthma [12,13]. Thymic stromal lymphopoietin (TSLP) was initially identified in the supernatant of a thymic stromal cell line. It has been demonstrated to boost B cell line growth and to encourage the proliferation of unfractionated thymocytes in response to anti-CD3 antibody stimulation [14]. Subsequently, it was shown to be an essential facilitator of type 2 immune responses and an inducer of illnesses mediated by T helper 2 (TH2) cells, such as asthma. Following up on this initial characterization, research has demonstrated that TSLP is implicated in a number of different illnesses and host responses in addition to its diverse functions in cell maturation, proliferation, survival, and recruitment [12]. It is now widely acknowledged that TSLP has a role in the development of pathological disorders, including cancer, host defence, allergy disease, and chronic inflammatory disease (Figure 1). TSLP is a type I cytokine composed of four α-helices and is a paralogue of IL-7 [15]. Although first shown to act on B cells, TSLP was then found to act directly on dendritic cells (DCs) and to indirectly affect T cells based on its effects on DCs [16]; furthermore, it was further demonstrated that TSLP directly affects CD4+ and CD8+ T cells and is necessary for the correct growth of CD4+ T cells. Moreover, group 2 innate lymphoid cells (ILC2s), smooth muscle cells, tumour cells, neutrophils, mast cells, basophils, and eosinophils are all impacted by TSLP [17,18]. The large variety of target cells in both people and mice explains the extensive range of actions that this cytokine can mediate. The lungs, skin, and gastrointestinal tract’s epithelial and stromal cells are the main producers of T lymphokine signalling protein (TSLP) in both homeostatic and inflammatory situations. Nevertheless, DCs, basophils, and mast cells can also secrete this cytokine. Many triggers, such as mechanical injury, ligands for Toll-like receptor 3 (TLR3), TLR2 and NOD2, helminth infection, pro-inflammatory cytokines, and proteases, such as trypsin and papain, can cause the formation of TSLP [19,20].

Viral infections, such as those caused by rhinovirus, influenza virus, respiratory syncytial virus (RSV), and lymphocytic choriomeningitis virus, can also cause the synthesis of TSLP in the lungs [21,22]. Inflammation is exacerbated by the fast release of TSLP from cells, which triggers additional endogenous and extrinsic danger signals. Tumour necrosis factor (TNF), IL-1β, IL-25, and the pro-inflammatory TH2-type cytokines IL-4 and IL-13 all upregulate the production of TSLP, and TNF works in concert with these cytokines to enhance TSLP synthesis. On the other hand, TSLP release is inhibited by IL-17 and interferon-γ (IFNγ) [21]. In addition to blocking TSLP release, β2-Adrenoceptor agonists and glucocorticoids work synergistically to block poly(I:C)-induced TSLP release [23]. Furthermore, mast cell synthesis of TSLP is induced by the cross-linking of IgE coupled to its high-affinity receptor (FcεRI) [24]. Nuclear factor-κB (NF-κB) and AP1 contribute to the transcriptional level of TSLP gene expression, and binding sites for NF-κB have been found in the TSLP promoter region [25]. Two forms of TSLP have been identified in humans: a long form (lfTSLP) and a short form (sfTSLP) [22]. The long form is also known as TSLP and is the molecule that corresponds to the murine one. With a total of 63 amino acids, as opposed to 159 for lfTSLP, sfTSLP’s transcription starts from a promoter in intron 2 and is, therefore, truncated at the amino-terminus but shares the same carboxy-terminus [22]. Keratinocytes, epithelial cells, and lung fibroblasts all express the short form of TSLP, in which inflammation has no upregulatory effect; on the other hand, the long form is induced by TLR ligands, including flagellin, and TNF [23,26]. Since these two forms have different regulatory mechanisms, it is clear that they perform different roles, with an antibacterial or anti-inflammatory function proposed for sfTSLP and a pro-inflammatory function for lfTSLP [24,25]. This idea was illustrated in a mouse asthma model study where the administration of sfTSLP improved asthma brought on by house dust mites, but the administration of lfTSLP weakened the function of the airway barrier, which may have contributed to the development of asthma [27]. The TSLP promoter contains single nucleotide polymorphisms (SNPs) rs2289276 and rs2289278 that enhance binding by AP1 and increase the production of lfTSLP. This mechanism may account for the association between these SNPs and an increased prevalence of adult asthma and childhood atopic illness [23]. Furthermore, it has been shown that the short form is more common in ovarian and endometrial malignancies and promotes tumour growth by triggering signalling pathways in cancer cells [28].

### 2.1. TSLP Signalling

**Figure 2:**
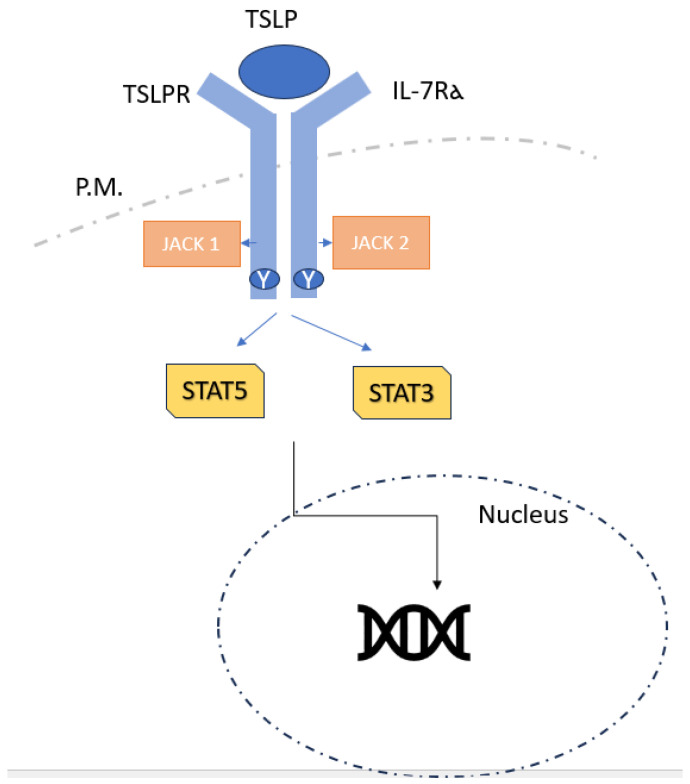
TSLP receptor.

TSLPR, a type I cytokine receptor encoded by Crlf2, and the IL-7 receptor α-chain (IL-7Rα; also called CD127) (Figure 2) form a heterodimeric receptor via which TSLP signals [29,30,31]. Target cells for TSLP include neurons, epithelial cells, mast cells, macrophages, basophils, and T cells. These cells also express receptors. TSLP stimulates JAK1 (by IL-7Rα) and JAK2 (via TSLPR), in contrast to its paralog IL-7, which activates JAK1 and JAK3 via a heterodimeric receptor made up of IL-7Rα and the common cytokine receptor γ-chain. After that, JAK1 and JAK2 mainly activate STAT5A, STAT5B, and, to a lesser extent, STAT1 and STAT3, which, in turn, promotes the production of IL-4, IL-5, IL-9, and IL-13 in addition to having pro-inflammatory effects [32,33]. Unlike lfTSLP, it is unknown if sfTSLP uses IL-7Rα and TSLPR together to signal, or if it uses a different signalling pathway. It is commonly known that TSLP and other cytokines generated from epithelial cells, IL-25, and IL-33 are essential in the development of allergic disorders, such as food hypersensitivity, asthma, and AD [34]. By acting on DCs and promoting the expression of OX40 ligand (OX40L), CD80, and CD86, TSLP promotes allergic reactions by stimulating the differentiation of naive CD4+ T cells into pro-inflammatory TH2 cells that generate IL-4, IL-5, IL-13, and TNF [35]. Additionally, it was demonstrated that TSLP-activated DCs encourage naive CD4+ T cells to develop into T follicular helper cells (expressed by CXCR5, IL-21, CXCL13, and BCL6), which can then trigger memory B cells to secrete IgG and IgE, establishing a connection between TSLP and IgE production in allergy development. Additionally, eosinophils, mast cells, and macrophages release TH2 cytokines and chemokines in response to TSLP [36,37]. The establishment of an IgE-independent mouse model of eosinophilic oesophagitis, a food-allergy-related inflammatory disease, has been connected with effects on basophils; however, the role of TSLP in basophil responses is yet unknown [38,39]. TSLP-driven type 2 immunity has been linked to a DC-T cell–basophil cascade, in which DCs activated by TSLP stimulate CD4+ T cells via OX40L to create IL-3, which, in turn, triggers the recruitment of basophils and the generation of IL-4 [40]. It has been shown that DCs themselves can increase the creation of TSLP, indicating that TSLP may also function autocrinally to enhance the TH2 cell response [41]. Since TSLP is necessary for CD4+ T cell complete proliferation in response to antigens, memory TH2 cell production, and recall responses, it also directly affects CD4+ T cells [42]. Crlf2−/− mice, who lack TSLPR, show robust TH1 cell responses correlated with high IFNγ, IL-12, and IgG2a levels, but low IgE, IL-4, IL-5, IL-10, and IL-13 levels. Crlf2−/− mice do not experience ovalbumin (OVA)-induced lung inflammation unless they are supplied with wild-type CD4+ T cells, and Crlf2−/−CD4+ T cells respond to antigen3 very weakly [27].

Interestingly, depending on whether TSLP is operating on innate or adaptive immune cells, it acts differently in models of airway inflammation.

### 2.2. TSLP and Respiratory Disease

One of the most relevant studies concerning the level of TSLP serum of COPD patients was published in February 2023 [43]. The authors measured serum levels of TSLP in 562 COPD patients to assess the exacerbation risk according to TSLP levels. In COPD patients, TSLP was higher and had a negative correlation with sputum eosinophil counts. TSLP may, therefore, potentially be connected to immunity that is not allergic. Cigarette smoke promotes the expression of TSLP in the epithelium, which may explain why some COPD patients have higher expression of TSLP. An intrinsic cytokine involved in both adaptive and allergic airway inflammation is called TSLP. The primary findings demonstrated that low eosinophil counts were the only group in which there was a significant correlation between TSLP level and decreased exacerbation risk. Additionally, TSLP level was linked to poor exercise ability. Authors concluded that TSLP could be a potential biomarkers of prognosis and a future target for use in target therapy in COPD patients [44]. In 2018, Yan Li et al. [45] analysed the expression of TSLP and IL-25 in asthma pathogenesis. The study was performed because it was known that alarmins promote Th2-type cytokine synthesis, but their expression was poorly documented in “real-life” human asthma. They assessed the quantities of these mediators in the airways and related them to lung function, neutrophil and eosinophil airway infiltration, and cytokines of the Th1 and Th2 types using bronchoalveolar lavage fluid (BALF) from seventy asthmatic patients. Asthmatics had significantly higher median BALF concentrations of IL-33, TSLP, IL-4, IL-5, IL-13, and IL-12p70 than controls (*p* < 0.05). Independent of corticosteroid medication, the concentrations of TSLP and IL-33 associated negatively with the forced expiratory volume in the first second (FTV) in asthmatics (TSLP: r = −0.565, *p* < 0.0001; IL-33: r = −0.488, *p* < 0.0001). The findings demonstrated the critical roles that TSLP and IL-33 play in the aetiology of asthma, which is typified by enduring airway inflammation and reduced lung function, indicating their potential as molecular targets [29]. From these considerations, new biological treatments were developed.

### 2.3. TSLP and Blood Neutrophil-to-Lymphocyte Ratio (NLR)

Neutrophils are essential in the chronic airway inflammation associated with bronchiectasis; but, in certain situations, lymphocytes, eosinophils, and other inflammatory cells have also been shown to be significant players. Furthermore, certain lymphocyte strains are essential for the immune system’s regulation of the inflammation associated with bronchiectasis. In this regard, studies on patients with bronchiectasis have examined the blood neutrophil-to-lymphocyte ratio (NLR); more precisely, the relationships between the NLR and severity and prognostic indicators have been examined. According to the currently used scores (FACED, E-FACED, and BSI), Martinez-García et al. (2022) [46] demonstrated that higher NLR was linked to more severe bronchiectasis as well as to worse quality of life (SGRQ), more comorbidities (Charlson index), and infection by pathogenic microorganisms. Furthermore, compared to other systemic inflammatory indicators, the NLR had a stronger correlation with severity levels. Lastly, it served as a separate predictor of both the frequency and intensity of exacerbations. In summary, the NLR was found to be a low-cost, simple-to-measure indicator of systemic inflammation that can be used to assess the severity of bronchiectasis patients and forecast exacerbations, particularly the most severe ones [30].

Lymphocytes represent adaptive immunity against both bacterial infection and the hyperexpression of neutrophilic inflammation, which can further damage the bronchial wall due to the proteolytic products secreted by neutrophils. Neutrophils play a critical role in the control of infection, both in terms of numbers and functions. Reductions in lymphocytes in circulation correspond with recent infections. Thus, via intricate processes, neutrophils and lymphocytes can also be cooperatively regulated. Other important analysis was performed to better understand the NLR role in respiratory disease.

Cataudella et al. [47] conducted a retrospective analysis to explore the performance of the neutrophil-to-lymphocyte ratio (NLR) to predict prognosis in a cohort of elderly adults with community-acquired pneumonia (CAP). The results encouraged early discharge of individuals with a NLR of less than 11.12 and admission to a respiratory intensive care unit for those with a NLR greater than 28.3 [31].

In 2022, Regolo et al. [48] provided evidence that NLR is an independent predictor of mortality and a worse outcome in COVID-19 patients and may help identify high-risk individuals with COVID-19 infection at admission [49].

The following year, the same author evaluated the relationships between NLR and CRP, as continuous variables, and the PaO_2_/FiO_2_ ratio (P/F) in severe cases of SARS-CoV-2 infections. NLR was demonstrated to be an independent predictor of mortality and significantly and directly linked to P/F, while the influence of inflammation on P/F, reflected by CRP, was also mediated by neutrophils [50].

Concerning the relationship between TSLP and the immune system, it is well known that there is a pleiotropic cytokine that acts on multiple cell lineages [51].

It has been demonstrated that TSLP improves neutrophil-mediated MRSA, killing when it directly interacts with neutrophils. Also, Streptococcus pyogenes, a significant contributor to skin infections, is more effectively eliminated by TSLP action. TSLP mediates its antibacterial activity by modulating neutrophil-produced reactive oxygen species, through direct engagement of the complement C5 system [51].

Moreover, it has been shown that TSLP mRNA and protein levels were increased in the bronchial epithelium of COPD compared with controls. Respiratory viruses, double-stranded RNA, cigarette smoke extracts, and pro-inflammatory cytokines activate NF-κB20, which, in turn, stimulates the production of TSLP in patients with COPD, suggesting involvement of TSLP in the development and/or exacerbation of COPD and, thus, that TSLP can affect lung pathophysiology in both TH1- and TH2-related diseases [51].

Starting from the above considerations, an analysis involving both NLR and TLSP levels in both exacerbation and clinical stability status could be helpful in improving knowledge on the pathogenetic role of inflammation and the dysfunction of the immune system in bronchiectasis.

### 2.4. Measurement of TSLP

Luminex-Multiplex kits (Komab biotech, Seoul, Republic of Korea) were used to assess the plasma levels of TSLP in the COPD patients in the investigation [43]. Within 30 min after being collected, blood samples were placed in tubes containing sodium ethylenediaminetetraacetic acid (EDTA) and centrifuged for 10 min at 1000× *g*. The plasma was kept between ≤−80 °C and processed using a customized Human Cytokine/Chemokine Magnetic Bead Panel (Millipore Corp., Billerica, MA, USA) in a 96-well plate. The authors followed the kit-specific protocol of the manufacturer of the Luminex 200analyzer (Luminex, Austin, TX, USA), which ran Master Plex QT software version 4 (MiraiBio, San Bruno, CA, USA). The manufacturer’s standards were used to create standard curves for each analyte. The authors used median values to differentiate between groups with high and low TSLP. The median TSLP level was 0.78 pg/mL; they formed TSLP-low and TSLP-high groups using this value as the cutoff [44].

## 3. Mucins

Mucociliary clearance is known to play a crucial role in bronchiectasis together with inflammation, infection, and structural damage; together, they are considered the pillars of the well-known “vicious circle” of this disease. In the emergent endotyping concept, it would be helpful to not only investigate the “4 pillars” but also any potential relationships between them. Mucociliary clearance is the mechanism in which secretions produced by respiratory cells entrap pathogens, removing them from the bronchial tree through coordinated cilia movement in bronchial epithelial cells. This important process is impaired in bronchiectasis, leading to the production of abundant secretions, potentially resulting in respiratory infections and/or colonisation by pathogens. Mucus is a protective coating secreted in the healthy airways, composed of water, salt, and proteins. The main macromolecular constituents of the mucus gel in healthy individuals are mucins. These glycoproteins oversee the mucus’s clearing and protecting qualities. Many mucins have been identified in the lower respiratory tracts of healthy people. However, the most extensively researched and major secreted mucins found in sputum are MUC5AC, MUC5B, and MUC2 [52]. The airway’s viscoelastic qualities depend on MUC5AC and MUC5B. Goblet cells, submucosal gland ducts, and the bronchial epithelium, to a lesser degree, all express MUC5B. It is produced in response to multiple stimuli, including inflammatory cytokines, like interleukin (IL) 1β, IL-6, IL-9, IL-13, and tumour necrosis factor (TNF) α, and possesses antibacterial capabilities [53]. Different host and environmental stressors, including proteases, bacteria, reactive oxygen species, air pollutants, cigarette smoke, cytokines, and airway compression, cause an increase in MUC5AC expression at the transcriptional level [35,54].

### 3.1. Mucins and Respiratory Diseases

The thin layer of liquid covering the respiratory tract’s luminal surface that is made up of component macromolecules is called airway mucus. Mucus’ primary job is to shield the lungs from harmful substances and foreign particles that are breathed by clearing the mucociliary system. In addition to water and ions, mucin glycoproteins and other macromolecules—some of which have antimicrobial, anti-protease, and antioxidant properties—make up mucus. Mucins are mostly glycoproteins that are secreted by cells and make up most of the protein content of mucus.

In the lung, MUC2, MUC5AC, MUC5B, and MUC19 are the four secreted mucins. The primary intestinal mucin, MUC2, is expressed by the colon’s and small intestine’s goblet cells. Its expression has also been noted in the lungs of sick people. The two main components of mucus gel in normal airways, MUC5AC and MUC5B, are thought to have a role in the barrier function of airway mucus.

The MUC5AC and MUC5B genes are in region p15.5 of chromosome eleven, which is also home to the MUC2 and MUC6 genes. These sequences overlap. The protein backbones of MUC5AC and MUC5B are multidomain structures with D, B, C, and CK domains on either side of central, O-glycosylated tandem repeats. MUC5AC might work by aiding in mucociliary clearance in general, while MUC5B might be particularly important in the removal of particular infections or irritants in the airways [55].

Mucins’ function in respiratory disorders was initially investigated in bronchiectasis associated with cystic fibrosis (CF); certain investigations revealed a lower level of secreted mucins in CF patients than in healthy individuals. Furthermore, concentrations of mucins rise (above all, MUC5AC) during a CF exacerbation, indicating a potential role for these molecules in these patients’ immune responses to inflammatory or infectious stimuli [56]. Recent experiments have verified that secreted mucins play a critical role in airway defence [57]. Nevertheless, there is a lack of information on the function of secreted mucins and their connection to bacterial airway infection in bronchiectasis.

Numerous authors proposed that elevated amounts of secreted mucins might be linked to bacterial colonization and the severity of the disease in bronchiectasis. As a result, in 2015, Sibila et al. [58] assessed mucin levels in patients with and without airway bacterial colonization who had bronchiectasis (not CF). This study has shown that patients with bronchiectasis and airway bacterial colonization—particularly those with *P*. *aeruginosa* colonization—had increased MUC2 levels in their sputum. While MUC5B levels were undetectable in most patients, there were no variations in MUC5AC levels between the groups.

Prominent levels of MUC2 and MUC5AC were associated with disease severity. These results lead to the hypothesis that mucins could have a role in bronchiectasis pathogenesis and, thus, in airway defence [52]. Fujisawa et al. [59] found, in 2015, that both IL-1β and IL-17A, two prominent pro-inflammatory cytokines associated with chronic airway inflammation, can mediate MUC5B induction in airway epithelial cells; despite this, persistence of inflammatory stimuli over time inhibits mucin production [60]. In 2020, Ramsey et al. [61] analysed mucin concentrations and their biochemical properties in non-cystic fibrosis bronchiectasis mucus. Their analysis showed that MUC5B, MUC5AC, and, to lesser extent, MUC2 were more expressed than in healthy subject [38].

It is now well established that COPD phenotypes are characterised by increased sputum production together with lung function and exacerbations. Consequently, current studies are analysing mucins as a potential marker of COPD patients’ health status; genetic variants associated with MUC5AC and MUC5B are supposed to be the most promising.

Another work published in January 2023 [53] highlighted a significant association with increased production of MUC5AC in severely asthmatic patients; they noted increased sputum production together with increased thickness of the airway walls and, consequently, a more severe form of asthmatic disease. Meanwhile, the MUC5B level was reduced in the same population, probably related to reduced mucociliary clearance [39].

Many conditions affecting the airways, such as asthma, CF, and COPD, cause an increase in MUC5AC. Patients with stable COPD have higher levels of MUC5AC expression in their bronchial submucosal glands [53]. MUC5AC expression was strongly elevated during allergic airway inflammation, while MUC5B expression stayed constant [62]. Using a quantitative Western blotting assay, Thornton and Sheehan (2004) [63] assessed the levels of MUC2, MUC5AC, and the various glycoforms of MUC5B in sputum. They found that CF and COPD patients’ sputum had higher levels of MUC5B, especially its low-charge glycoform, than did the secretions of normal subjects and asthmatic subjects.

While MUC5B is thought to be more associated with COPD and cystic fibrosis, MUC5AC production is more likely to be relevant to the pathophysiology of asthma [40].

Furthermore, research conducted in 2004 by Thornton and Sheehan [63] showed that varying levels of MUC5AC and MUC5B were present in airway mucus, even in healthy persons. This variation could be caused by variations in the genetic polymorphisms of the corresponding mucin genes. Furthermore, the association between pulmonary fibrosis and a polymorphism in the promoter of the MUC5B gene is now well established [64].

Normal lung function depends on the creation and control of mucus. Since pathogenic agents and host inflammatory mediators trigger mucin gene expression in chronic lung disorders, mucus overproduction is closely correlated with the morbidity and mortality of airway diseases (the most significant being chronic obstructive pulmonary disease, or COPD, cystic fibrosis, and asthma).

Goblet cell metaplasia (GCM), the reversible transformation of non-goblet airway epithelial cells into goblet cells, and goblet cell hyperplasia (GCH), which refers to increasing goblet cell populations, are additional mechanisms by which microbial pathogens and host response molecules promote airway remodelling. Both pathogenic processes lead to mucus blockage of the airways and maintain an excess of mucin in the airways. It is crucial to remember that, despite any clinical connections, GCH and GCM originate from different cellular and molecular processes that might or might not be connected to the overproduction of mucin.

It is unknown what exact processes control GCH and GCM. Effective treatment of airway disorders originating from mucus overproduction will only be realized once the complexity of these routes and processes are entirely understood [55].

For the ciliary clearing system to work normally, mucus and cilia are both essential components, and abnormalities in any one of these elements can result in serious illness and airway dysfunction.

These controversial data raised interest in mucin’s role in all chronic respiratory diseases characterised by increased sputum production and is currently a major topic under investigation.

Mucin analysis of bronchiectasis patients’ sputum could have a role in endotyping. It has been demonstrated in other chronic lung diseases that elevated levels of mucins are associated with increased sputum secretion and more enlarged airways. Therefore, their dosage could have a relevant role in disease severity stratification.

### 3.2. Measure of Mucins on Sputum

In many studies that have been previously published (such as the one conducted by Sibila in 2015 [58]), sputum was centrifuged at 5000× *g* for 10 min at 10 °C. The solid phase was removed and cooled at −70 °C. Proteases inhibitors (Calbiochem, San Diego, CA, USA) were added (at equal volume) to sputum samples during defrosting. Mucins MUC2, MUC5AC, and MUC5B were measured using an ELISA kit and commercially validated (USCN Life Science Inc., Wuhan, China) [52].

## 4. Conclusions

A vicious cycle of airway infection, inflammation, reduced mucociliary clearance, and structural damage leading to disease development characterize the pathogenesis of bronchiectasis. The pathophysiological pathways (endotypes) and underlying molecular causes of chronic respiratory disease have received more attention in recent years. We know from the literature that in bronchiectasis these features can vary considerably. Focusing on inflammatory endotypes will allow us to identify new potential biomarker and, consequently, provide increasingly patient specific personalised therapy. Pleiotropic cytokine TSLP is implicated in cancer development, chronic inflammation, fat metabolism, and viral infections such as SARSCoV-2 and influenza viruses, apart from their well-known role in type 2 inflammatory responses. Given the circumstances, TSLP’s functions in cellular homeostasis and a variety of disorders point to its potential as a therapeutic target and predictive marker of disease severity. Consequently, TLSP serum (or on sputum/bronchoalveolar lavage) could be promising in bronchiectasis to establish if there is a correlation between its level, disease severity, and, eventually, clinical outcomes. Moreover, bronchiectasis is a chronic respiratory disease characterized by an underlying inflammatory status; therefore, new biological markers could provide more information on pathogenesis. Excessive mucus production and reduced mucociliary clearance are “pillars” of bronchiectasis, as stated above; hence, mucin analysis of bronchiectasis patients’ sputum could be fundamental for discovering the endotypes of disease. As demonstrated in other obstructive airways diseases, mucin dosage could have a role in risk stratification and the severity of bronchiectasis. Knowledge of the inflammatory pathway is constantly increasing because of this developing awareness. To identify targets for potential future therapies, it is imperative to continue characterizing the primary inflammatory drivers in various cohorts of patients with bronchiectasis. It is essential for future studies to highlight these underlying mechanisms, which might be responsible for the variation in medication responses. A better comprehension of underlying inflammatory drivers through non-invasive analysis methods, such as those used for detecting peripheral biomarkers, is of immense importance for disease management. Biomarkers need to be associated with the clinical, radiological, and functional status of the disease to acquire significance. Finally, starting from the evidence outlined by research data, we might develop future clinical trials that involve new inflammatory markers as target therapy, both related to selective cytokine inhibition and to new mucolytic drugs.

## Figures and Tables

**Table 1:** Genetic variants in bronchiectasis.

Genetic Variant	Comment
MBL deficiency	Mannose-binding lectin (MBL) is a key component of innate immunity involved in the clearance of bacteria and apoptotic cells. Genetic MBL deficiency is common in the general population and related to disease severity in bronchiectasis, including quality of life and frequency of exacerbations and admission to hospital.
Telomere	Telomere attrition is an established ageing biomarker. Lim et al. [4] reported that shortened telomere length was significantly relevant in sputum immune cells of bronchiectasis patients.
GBP5	GBP5 upregulation, a positive regulator of the NLRP3 inflammasome, led to exaggerated immune response upon bacterial infections.
FUT2	Secretion of a (1,2) fucosylated glycans elicits a dichotomous effect on host–microbe interactions, making the secretor genotype (FUT2) a risk factor underlying variation in infection type and disease severity in bronchiectasis. Homozygous secretors exhibit lower lung function, higher exacerbation rate, and more frequent *P*. *aeruginosa*-dominated infection
CFTR	Mutations associated with the following phenotypes: - Cystic fibrosis; - Congenital bilateral absence of vas deferens; - Sweat chloride elevation without CF; - Bronchiectasis.
DAW1	Motile ciliopathy laterality disorder.
DNAH5	Mutations associated with the following phenotypes: - Bronchiectasis; - Primary ciliary dyskinesia; - Ciliary dyskinesia with or without situs inversus.
PIK3CD	Immunodeficiency; autosomal dominant
SCNN1A	Mutations associated with the following phenotypes: - Pseudo hypoaldosteronism; - Bronchiectasis with or without elevated sweat chloride.
SCNN1B	Mutations associated with the following phenotypes: - Non-classic cystic-fibrosis-like syndrome; - Liddle syndrome; - Bronchiectasis.
SCNN1G	Mutations associated with the following phenotypes: - Liddle syndrome; - Pseudo hypoaldosteronism, type I; - Bronchiectasis
DNAI1	Mutations associated with the following phenotypes: - Bronchiectasis; - Ciliary dyskinesia.
DNAI2	Mutations associated with the following phenotypes: - Bronchiectasis; - Primary ciliary dyskinesia.
DNAL1	Mutations associated with the following phenotypes: - Bronchiectasis; - Ciliary dyskinesia.
RSPH4A	Mutations associated with the following phenotypes: Bronchiectasis with rhinorrhoea, rhinitis, nasal blockage, sinusitis, otitis media, hearing problems, deafness, low weight, and short stature.
PCDHGA7 and PCDHGB4	Members of the protocadherin-γ gene cluster; decreased expression may be related to airway dilation in early bronchiectasis.
Proteasome 20S subunit-β (PSMB)five	The immunoproteasome has been shown to enhance the generation of major-histocompatibility-complex-I-associated peptide and facilitate the antiviral immune response in the lung. Furthermore, IFN-γ has previously been shown in mice to play an essential role in protective immunity against tuberculosis and mycobacterial infection, both of which are relevant to infection in bronchiectasis airways.
Immunoproteasome subunits PSMB8, PSMB9, PSMB10	The immunoproteasome has been shown to enhance the generation of major-histocompatibility-complex-I-associated peptide and facilitate the antiviral immune response in the lung. Furthermore, IFN-γ has previously been shown in mice to play an essential role in protective immunity against tuberculosis and mycobacterial infection, both of which are relevant to infection in bronchiectasis airway.
LGR5	Highly expressed in basal cells; may alter the structure of the airway or the ability to repair damaged airway epithelium.
Wnt signalling pathway	Wnt signalling pathway activity decreases with age and may explain age-associated severity in bronchiectasis.
SERPINA1	Association between these genetic variants and α1-antitrypsin deficiency, which can lead to multiple pulmonary pathologies including chronic bronchitis and bronchiectasis.
DEUP1	High expressions of DEUP1, FOXN4, and CDC20B, have been reported as precursors of multiciliated cells. A hypothesis is that the overproduction of certain ciliary proteins contributes to faulty cilia assembly, reduced clearing capacity of the lung, and bronchiectasis pathogenesis.
FOXN4	High expressions of DEUP1, FOXN4, and CDC20B, have been reported as precursors of multiciliated cells. A hypothesis is that the overproduction of certain ciliary proteins contributes to faulty cilia assembly, reduced clearing capacity of the lung, and bronchiectasis pathogenesis.
CDC20B	High expressions of DEUP1, FOXN4, and CDC20B, have been reported as precursors of multiciliated cells. A hypothesis is that the overproduction of certain ciliary proteins contributes to faulty cilia assembly, reduced clearing capacity of the lung and bronchiectasis pathogenesis.
